# Novel scoring system to predict futile liver transplantation by multiterm outcomes to optimize recipient selection: retrospective cohort study

**DOI:** 10.1093/bjsopen/zraf108

**Published:** 2025-10-15

**Authors:** Xi Wang, Xiubi Yin, Shaohua Song, Di Jiang, Yuancheng Li, Zeliang Xu, Xingchao Liu, Zhu Li, Xiaofang Zhang, Chengcheng Zhang

**Affiliations:** Department of Hepatobiliary Surgery, Southwest Hospital, Third Military Medical University (Army Medical University), Chongqing, China; Department of Hepatobiliary Surgery, Southwest Hospital, Third Military Medical University (Army Medical University), Chongqing, China; Department of General Surgery, Ruijin Hospital, Shanghai Jiao Tong University, School of Medicine, Shanghai, China; Department of Hepatobiliary Surgery, Southwest Hospital, Third Military Medical University (Army Medical University), Chongqing, China; Department of Hepatobiliary Surgery, Southwest Hospital, Third Military Medical University (Army Medical University), Chongqing, China; Department of Hepatobiliary Surgery, Southwest Hospital, Third Military Medical University (Army Medical University), Chongqing, China; Department of Hepatobiliary Surgery, Sichuan Academy of Medical Sciences and Sichuan Provincial People’s Hospital, Chengdu, China; Department of Organ Transplantation Surgery, Liaocheng People’s Hospital/Affiliated Liaocheng Hospital, Shandong First Medical University, Liaocheng, China; Department of Hepatobiliary Surgery, Southwest Hospital, Third Military Medical University (Army Medical University), Chongqing, China; Department of Hepatobiliary Surgery, Southwest Hospital, Third Military Medical University (Army Medical University), Chongqing, China

**Keywords:** individualized prediction, futile scoring system

## Abstract

**Background:**

Improvements in medical standards have allowed critically ill patients to benefit from liver transplantation, but defining futility arbitrarily according to one single-stage outcome could deprive patients of the potential benefits of transplantation. This study aimed to redefine futile liver transplantation by multiterm outcomes and develop a novel scoring system to predict futile liver transplantation.

**Methods:**

This retrospective study in China enrolled patients who had liver transplantation from 3 centres between January 2015 and April 2021. Independent risk factors were identified by logistic regression analysis and used to establish risk prediction models. Kaplan–Meier survival curves were calculated to explore the association between futile score and overall survival.

**Results:**

Of 1408 patients undergoing liver transplantation, patients at persistent high risk for mortality in the short term (3 months), mid term (1 year), and long term (3 years) were defined as the truly futile liver transplantation group. Higher donor and recipient age, hepatorenal syndrome, intensive care unit stay, need for mechanical ventilator, ABO blood group incompatibility, prolonged cold ischaemia time, increased alanine aminotransferase levels, and decreased albumin levels were independent risk factors for futility, and were used to construct a futile scoring system. The scoring system had good predictive capability, with an area under the receiver operating characteristic curve of 0.921, better than that of a previously established scoring system. Survival analysis showed that the group with a high futile risk had decreased survival.

**Conclusion:**

This study has redefined futile liver transplantation and established a novel futile scoring system. This can be used to optimize the allocation of medical resources, especially with regard to recipient selection for liver transplantation, and increase survival prediction for selected patients.

## Teaser

This study redefined patients at persistent high risk as the truly futile liver transplantation group, and constructed a futile scoring system that combined recipient and donor factors. The scoring system had good predictive ability, and can be used to optimize the allocation of medical resources, especially with regard to recipients withdrawing from liver transplantation, and ensure the survival chances of certain patients.

## Introduction

Liver transplantation (LT) remains a vital life-saving tool for patients with end-stage liver disease^1^. Similar to cancer surgery, the benefits and the need to perform LT are determined by survival outcomes^2,3^. Advances in surgery, medical technology, and drug innovations have helped many critically ill patients who would not expect to gain short-term benefits from LT to achieve long-term survival after recovering from surgery^4^. In this situation, defining futile LT based on a single stage is not accurate enough, as mortality risk factors can vary temporally^5–7^. Therefore, it is essential to take temporal variation into consideration to identify true futility and to explore the risk factors leading to futile transplantation in order to avoid organ wastage and not deprive some patients of the survival benefit of LT.

Indeed, even at a single stage, the definition of futile LT elicits substantial controversy. A Delphi panel, comprising 35 multidisciplinary international experts, previously came to a consensus that established mortality within 1 year after LT as the benchmark for defining futility^8^. However, following meticulous investigation, Rolak *et al*.^9^ defined LT after which death occurred within 3 months as futile, both from the perspective of patients and their families and in terms of organ utility. It should be noted that the risk factors considered in these studies were not consistent. Therefore, patients’ eligibility for LT should remain unaffected unless the prognosis is clearly inferior to the desired threshold.

The mortality risk after LT is complex and changing dynamically. Despite some overlap, there are still discernible differences between short- and long-term mortality patterns and associated risk factors after LT^10,11^. In the short term, risk factors for death predominantly include surgical complexity with intraoperative massive haemorrhage or postoperative massive transfusion, individual patient conditions, acute rejection, and infection^11–14^. However, as patients progress into a long-term survival stage, some new risk factors emerge, including chronic complications, recurrent liver disease, transplant dysfunction, and non-adherence to medication^4,10,15^. The dynamic changes in risk factors have led to limitations in defining futility based on a single stage. Therefore, multiterm and dynamic monitoring and evaluation methods should be adopted. Until now, however, few studies have investigated the dynamic changes in mortality risk after LT, and there is a need to develop more efficacious models to more accurately identify truly futile LT.

To fill these gaps in defining true futility and associated risk factors, the authors adopted strict inclusion and exclusion criteria to construct survival models at different stages after LT, for tracking high-risk populations by stage. The aim of this study was to redefine futile LT by multiterm survival outcomes and to develop a novel scoring system to predict truly futile LT. Accurate definition of futility could optimize the allocation of medical resources, particularly in terms of recipient selection for LT, and improve the survival benefits of affected patients.

## Methods

### Study design and patient selection

This study enrolled adult patients with LT (aged ≥ 18 years) from Southwest Hospital and Sichuan Provincial People’s Hospital from January 2015 to April 2021. All donor and recipient data were recorded in the Chinese Liver Transplantation Registry (https://cltr.cotr.cn), and organs were derived only from deceased donors. All donor organs were sourced exclusively through voluntary, informed consent documented via IRB-approved consent forms signed either by the donor (pre-mortem autonomy) or their legally authorized next of kin (posthumous representation). Organs sourced from individuals subjected to capital punishment were explicitly excluded from this study. Patients with combined transplantation, those with incomplete clinical data, and patients lost to follow-up were excluded. This study conformed to the Helsinki Declaration and institutional ethics guidelines, and was approved by the ethics committee of Southwest Hospital (ethics approval number (B)KY2021013). Informed consent was waived owing to the retrospective design. This retrospective study is reported according to the Strengthening the Reporting of Cohort, Cross-sectional, and Case–Control Studies in Surgery criteria.

### Characteristic collection and processing

Demographic characteristics, pretransplant life support treatment, and laboratory data collected within 3 days before LT were used to identify features and construct survival models. Patient characteristics were collected as follows: donor age, donor sex, donor body mass index (BMI), donor type categorized as donation after circulatory death (DCD) or donation after brain death, recipient age, recipient sex, recipient BMI, Model for end-stage Liver Disease (MELD) score, balance-of-risk (BAR) score, hypertension, diabetes mellitus, reason for transplantation, hepatorenal syndrome (HRS)^16^, presence of malignancy, portal vein thrombus, previous transjugular intrahepatic portosystemic shunt (TIPS), previous abdominal surgery, hepatic encephalopathy (grade > 2), intensive care unit (ICU) stay before LT, mechanical ventilation before LT, artificial liver support before LT, ABO blood type incompatibility, and cold ischaemia time (CIT). Laboratory test results, including white blood cell (WBC) count, platelet count, levels of alanine aminotransferase (ALT), aspartate aminotransferase, albumin, plasma sodium, and total bilirubin, and prothrombin time international normalized ratio (INR) were recorded.

### External validation

For external validation, an external data set consisting of adult patients who underwent LT at Huashan Hospital in Shanghai from April 2015 to April 2021, was used. The inclusion and exclusion criteria were the same as those for the derivative cohort.

### Definitions of survival groups

Strict data cleaning procedures were implemented, with exclusion of patients who had died at the previous stage in each group, and three survival groups were ultimately constructed: short-, mid-, and long-term survival groups. The short-term survival group encompassed the entire study population, whereas the mid-term group comprised patients surviving beyond 3 months after LT (excluding those who died within 3 months after transplantation). The long-term survival group referred to individuals who were alive 1 year after LT (excluding patients who died within 1 year after transplantation).

### Development of survival models and risk stratification

This study adopted a phased modelling strategy. Univariable and multivariable logistic regression analyses were used to identify independent risk factors at different survival stages based on predefined survival groups, and short-, mid-, and long-term survival models were developed. Receiver operating characteristic (ROC) curves were plotted to evaluate the predictive performance of the three survival models. The optimal cut-off value was determined by maximizing the Youden index in the corresponding survival models. Subsequently, the total patient population was divided into high- and low-risk categories based on their optimal cut-off value at each stage, and systematically arranged and combined to form eight subgroups with time-varying risk characteristics.

### Redefinition of futile LT

To further validate truly futile LT, a hierarchical validation framework was proposed. The first level of validation assumed that the subgroups that had undergone two or more high-risk stages were considered as the futile candidate group, whereas the second level shrank the validation scope to subgroups that had sustained the high risk throughout the entire three stages (short term, medium term, long term) as the futile candidate group. To confirm the survival benefit of the two futile candidate groups, a dual validation mechanism based on Kaplan–Meier survival curves was adopted, with internal validation being conducted in the model-derived cohort, and cross-centre external validation in the external validation cohort. Finally, the futile candidate group with the lowest survival benefit in both the internal and external cohorts was identified as the truly futile LT group.

### Statistical analysis

Continuous variables are presented as mean(standard deviation) and were compared with Student’s *t* test. Categorical variables are presented as frequencies and percentages, with analysis by means of the χ^2^ or Fisher’s exact test. Univariable and multivariable logistic regression analyses were used to identify independent risk factors at different survival stages and to construct three survival models by stage. Similarly, after confirming the futile LT group, univariable and multivariable logistic regression analysis was used to screen for independent predictors for futility, and to construct a futile model. To evaluate the predictive performance of the futile scoring system, ROC curves were plotted, and areas under the curve (AUCs) were calculated in the derivative and validation cohorts. The optimal cut-off value was determined by maximizing the Youden index in the derivative cohort, and dividing the derivative and validation cohorts into groups with high and low futile risk based on this. In addition, to verify the consistency of the model, calibration plots were constructed. The decision curve analysis (DCA) method was used to comprehensively evaluate the application value of the established model in clinical practice by displaying the net benefit under different risk threshold probabilities. Kaplan–Meier survival curves were plotted for survival analysis. Two-tailed *P* < 0.050 was considered statistically significant. Data analyses were conducted in R 3.6.3 (R Foundation for Statistical Computing, Vienna, Austria) and SPSS^®^ version 26.0 (IBM, Armonk, NY, USA).

## Results

### Baseline patient characteristics

**Fig. 1:**
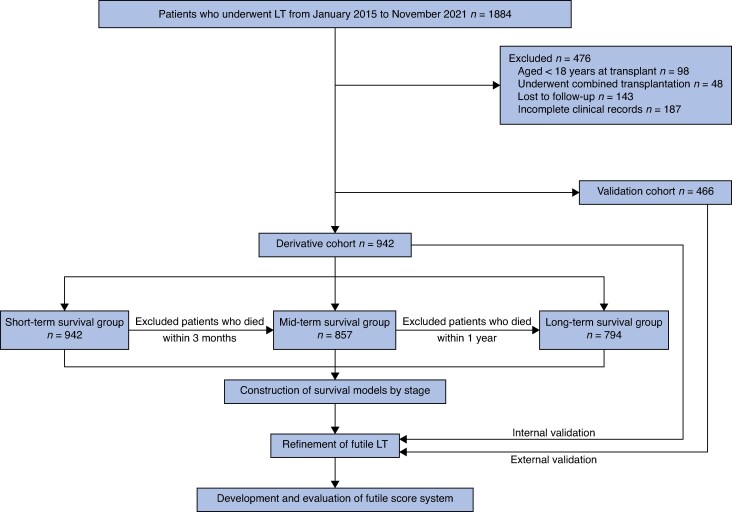
Study flow chart LT, liver transplantation.

A total of 1408 patients (942 in derivative cohort, 466 in validation cohort) who received LT were included in this study. Three survival groups were defined: short term (3 months, 942 patients), mid term (1 year, 857), and long term (3 years, 794) in the derivative cohort (*Fig. 1*). Patient characteristics and surgery-related parameters are summarized in *Table 1*. In terms of pretransplant life support, 207 patients (14.7%) were observed in the ICU before transplantation, and 52 (3.9%) were on mechanical ventilation. In addition, 141 patients (10.0%) received artificial liver support. There were no significant differences in demographic characteristics, pretransplant life support treatment, and laboratory data between the two cohorts.

**Table 1:** Baseline characteristics of patients

	Derivative cohort (*n* = 942)	Validation cohort (*P* = 466)	P*
Donor age (years), mean(s.d.)	46.65(13.05)	47.80(11.86)	0.107†
Donor BMI (kg/m^2^), mean(s.d.)	23.46(2.87)	23.45(2.84)	0.950†
**Donor sex**			0.461
Male	763 (81.0%)	385 (82.6%)	
Female	179 (19.0%)	81 (17.4%)	
Recipient age (years), mean(s.d.)	49.65(10.65)	50.08(10.49)	0.478†
Recipient BMI (kg/m^2^), mean(s.d.)	23.27(3.27)	23.41(4.00)	0.475†
**Recipient sex**			0.649
Male	771 (81.8%)	386 (82.8%)	
Female			
**Reason for transplantation**			0.079
HBV/HCV	515 (54.7%)	247 (53.0%)	
Alcoholic	266 (28.2%)	116 (24.9%)	
Autoimmune	48 (5.1%)	37 (7.9%)	
Other diseases	113 (12.0%)	66 (14.2%)	
Malignancy	386 (41.0%)	195 (41.8%)	0.755
Hypertension	104 (11.0%)	44 (9.5%)	0.364
Diabetes mellitus	99 (10.5%)	47 (10.1%)	0.806
Portal vein thrombus	35 (3.7%)	16 (3.4%)	0.790
Previous TIPS	88 (9.3%)	38 (8.2%)	0.463
Previous abdominal surgery	106 (11.3%)	56 (12.0%)	0.672
Hepatic encephalopathy	41 (4.4%)	28 (6.0%)	0.176
Hepatorenal syndrome	68 (7.2%)	32 (6.9%)	0.809
ICU stay	148 (15.7%)	59 (12.7%)	0.128
Mechanical ventilator	30 (3.2%)	22 (4.7%)	0.150
Artificial liver support	94 (10.0%)	47 (10.1%)	0.950
ABO incompatibility	202 (21.4%)	82 (17.6%)	0.090
**Type of donor**			0.974
DCD	260 (27.6%)	129 (27.7%)	
DBD	682 (72.4%)	337 (72.3%)	
Cold ischaemia time (h), mean(s.d.)	6.03(1.98)	6.07(1.28)	0.694†
MELD score, mean(s.d.)	22.48(10.94)	21.05(19.18)	0.075†
BAR score, mean(s.d.)	8.81(5.61)	8.64(3.45)	0.550†
**Pretransplant laboratory data**			
WBC (×10^9^/l), mean(s.d.)	5.58(3.69)	5.96(4.03)	0.073†
Platelets (×10^9^/l), mean(s.d.)	89.49(75.80)	92.83(45.7)	0.381†
ALT (units/l), mean(s.d.)	82.99(130.61)	86.81(150.51)	0.624†
AST (units/l), mean(s.d.)	105.8(140.68)	107.51(68.85)	0.804†
Albumin (g/l), mean(s.d.)	35.64(7.10)	35.08(4.41)	0.119†
Total bilirubin (mg/dl), mean(s.d.)	7.41(8.41)	7.5(8.15)	0.845†
Plasma sodium (mmol/l), mean(s.d.)	138.23(5.44)	138.77(6.03)	0.090†
Prothrombin time INR (s), mean(s.d.)	2.14(0.69)	2.17(1.13)	0.481†

Values are *n* (%) unless otherwise stated. s.d., Standard deviation; BMI, body mass index; HBV, hepatitis B virus; HCV, hepatitis C virus; TIPS, transjugular intrahepatic portosystemic shunt; ICU, intensive care unit; DBD, donation after brain death; DCD, donation after circulatory death; MELD, Model for End-stage Liver Disease; BAR, balance of risk; WBC, white blood cell; ALT, alanine aminotransferase; AST, aspartate aminotransferase; INR, international normalized ratio. *χ^2^ or Fisher’s exact test, except †Student’s *t* test.

### Changes in risk factors in relation to post-LT stage

Univariable and multivariable logistic regression analyses in the short-term survival group showed that higher recipient age, previous TIPS, HRS, ICU stay, need for mechanical ventilator, artificial liver support, ABO blood type incompatibility, prolonged CIT, MELD score, and BAR score were independent risk factors associated with short-term mortality (*Table S1*). Similarly, analysis in the mid- and long-term survival groups revealed that malignancy, hepatic encephalopathy grade > 2, prolonged CIT, increased ALT, total bilirubin and INR levels, and decreased albumin level were independent risk factors associated with mid-term mortality (*Table S2*); and higher donor age, higher recipient age, lower recipient BMI, malignancy, ABO incompatibility, DCD, prolonged CIT, and increased WBC and total bilirubin levels were independent risk factors associated with long-term mortality (*Table S3*). The results of logistic regression analysis clearly showed that there were significant differences in the risk factors affecting mortality among the different post-LT survival stages.

### Development and validation of survival models follow post-LT stage

Corresponding short-, mid- and long-term survival models in the predefined survival groups were then constructed based on risk factors at different stages after LT, accompanied by ROC curves assessing the predictive accuracy of the models. ROC curves had AUCs of 0.876, 0.823, and 0.808 for the short-, mid- and long-term survival models in the derivative cohort, and 0.855, 0.747, and 0.803 respectively in the validation cohort, demonstrating good predictive ability of the survival models (*Figs S1a* and *S2a*). Calibration curves were constructed using the bootstrap method for internal validation to assess the agreement between predicted and observed outcomes. The calibration plots showed good agreement between the predicted and observed probabilities in the derivative and validation cohorts (*Figs S1b* and *S2b*). DCA revealed that the models had a higher net benefit (*Figs S1c* and *S2c*). Subsequently, the three survival groups were segregated categorically into high- and low-risk groups. To validate the clinical applicability of the models, Kaplan–Meier survival curves were plotted for the high- and low-risk groups, which revealed statistically significant differences in survival benefit between the two groups across the three survival stages in the derivative and validation cohorts (*Figs S1d* and *S2d*).

### Changes in mortality risk in relation to post-LT stage

**Fig. 2:**
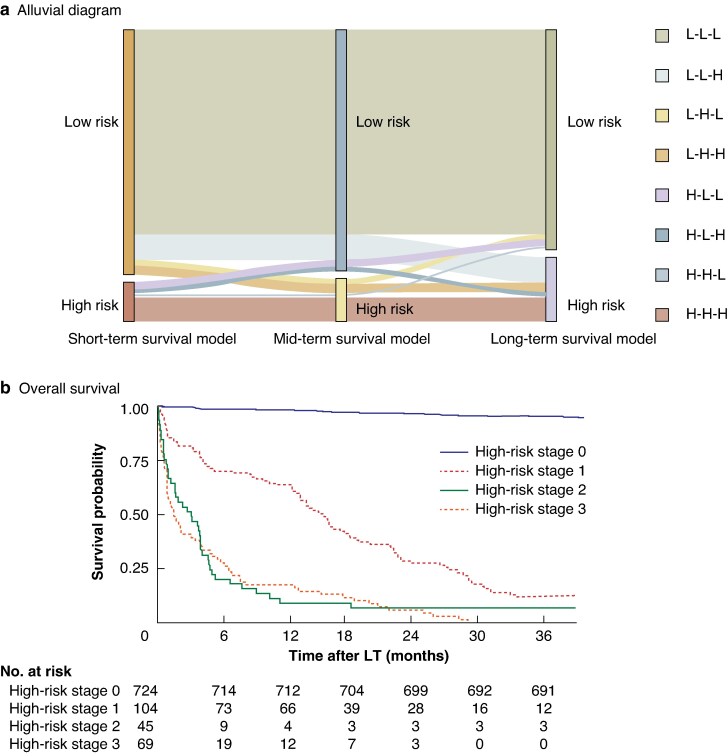

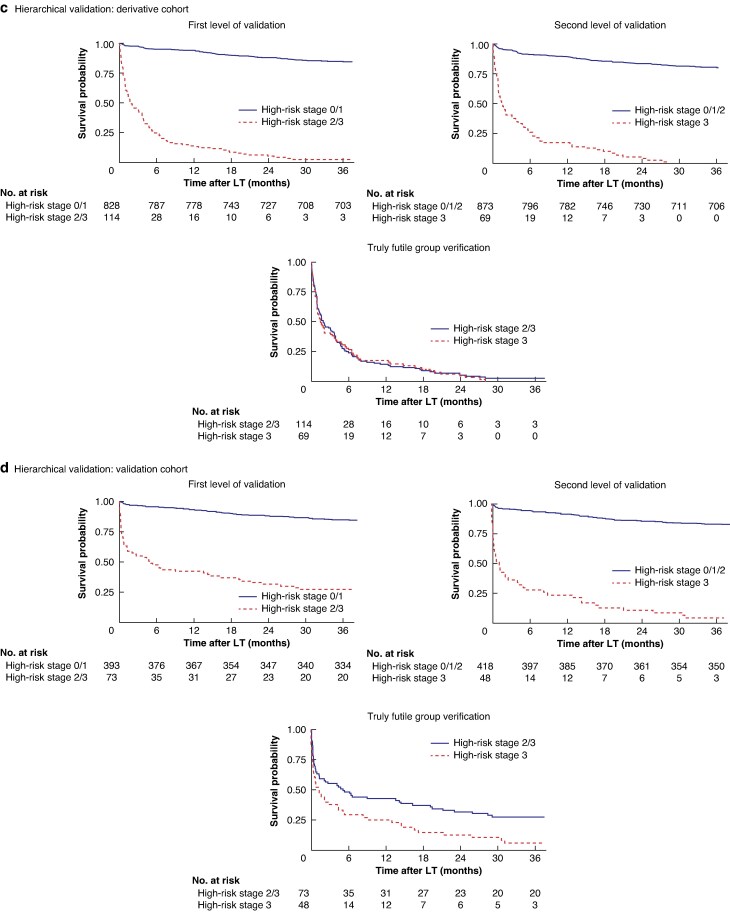
Alluvial diagram and survival analysis **a** Alluvial diagram for dynamic changes in mortality risk after liver transplantation (LT). L, low; M, medium; H, high. **b** Overall survival according to high-risk stages in derivative cohort. **c** Hierarchical validation of truly futile LT group in derivative cohort. **d** Hierarchical validation of truly futile LT group in validation cohort. *P* < 0.001 for all hierarchical validations, except *P* = 0.780 for truly futile group verification in derivative cohort (log rank test).

To obtain the risk distribution for the same patient at different survival stages, the overall patient population was divided into high- and low-risk groups by stage based on the optimal cut-off values for the three survival models. Based on the aforementioned risk stratification, an alluvial diagram was generated that demonstrated the dynamic changes in the risk stratification of patients after LT (*Fig. 2a*). Subsequently, the eight subgroups with time-varying risk characteristics were divided into four levels based on the number of high-risk stages, and corresponding Kaplan–Meier survival curves were plotted. After Bonferroni correction, patients with two or more high-risk stages after LT seemed to have the worst prognosis, whereas those with no high-risk stages after transplantation had the best prognosis (*Fig. 2b*).

### Refining futile LT

Based on risk stratification and survival analysis, the first level of validation was undertaken, assuming that the subgroups that have undergone two or more high-risk stages (high-risk stage 2/3) comprise the futile candidate group. Kaplan–Meier survival curves were plotted in the derivative cohort and external validation cohort for internal and external validation. The results showed that the survival benefit of the first-level validation futile candidate group was poor (*P* < 0.001). Subsequently, a second level of validation was conducted, assuming that the sustained high-risk subgroup (high-risk stage 3; H-H-H in *Fig. 2a*) was the futile candidate group. Kaplan–Meier survival curves were again plotted in the derivative cohort and external validation cohort for internal and external validation. The results showed that the survival benefit of the second-level validation futile candidate group was poor (*P* < 0.001). By further defining the truly futile group, survival analysis was conducted on two futile candidate groups in the derivative cohort and external validation cohort; it was found that the survival benefit of the second-level validation futile candidate group was the worst (*Fig. 2c*,*d*). Therefore, patients who were persistently at high risk were redefined as the truly futile LT group. The reasons for futility were infectious complications in 29 patients (42.0%), graft failure in 14 (20.3%), vascular complication in 8 (11.6%), multiple organ failure in 6 (8.7%), and unknown causes in 12 (17.4%) (*Fig. S3*).

### Development and validation of futile scoring system

**Fig. 3:**
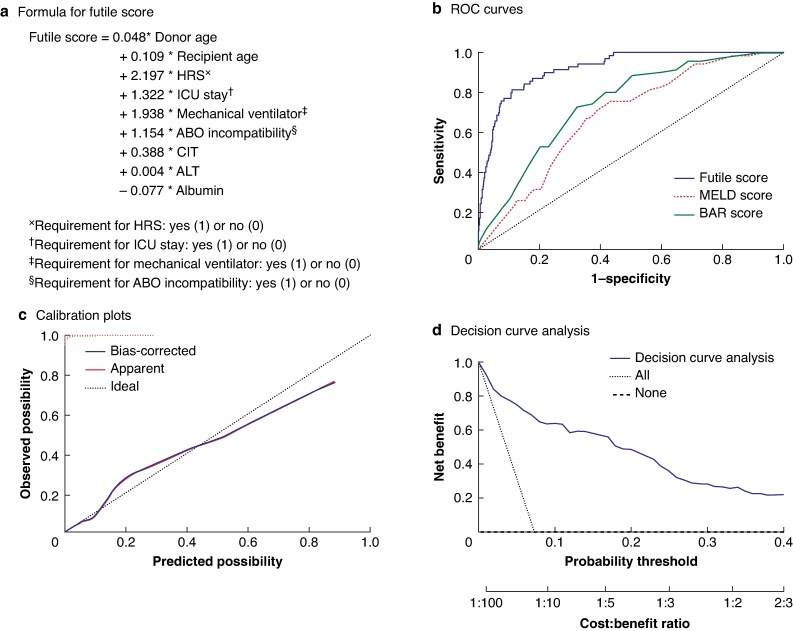
Development and validation of futile scoring system in derivative cohort **a** Formula used to calculate futile score. HRS, hepatorenal syndrome; ICU, intensive care unit; CIT, cold ischaemia time; ALT, alanine aminotransferase. **b** Receiver operating characteristic (ROC) curves for futile score (area under curve (AUC) 0.921, 95% confidence interval 0.893 to 0.949), Model for End-stage Liver Disease (MELD) score (AUC 0.680, 0.622 to 0.737), and balance-of-risk (BAR) score (AUC 0.742, 0.688 to 0.796). **c** Calibration curve for futile score for futility (B = 100 repetitions, boot. Mean absolute error 0.011; *n* = 942). **d** Decision curve analysis for futile score.

**Fig. 4:**
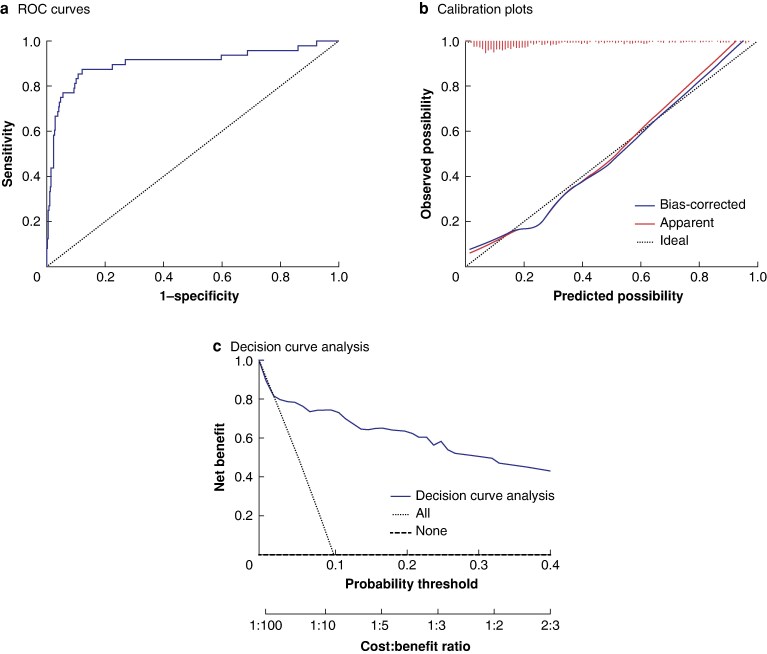
Validation of futile score system in validation cohort **a** Receiver operating characteristic (ROC) curve for futile score (area under curve 0.901, 95% confidence interval 0.840 to 0.963). **b** Calibration curve for futile score for futility (B = 100 repetitions, boot. Mean absolute error 0.027; *n* = 466). **c** Decision curve analysis for futile score.

Univariable and multivariable logistic regression analysis in the futile group showed that higher donor age, higher recipient age, HRS, ICU stay, requirement for mechanical ventilator, ABO incompatibility, prolonged CIT, increased ALT level, and decreased albumin level were the independent risk factors associated with futile outcomes (*P* < 0.005) (*Table 2*). Based on the bootstrap estimates of the final logistic regression model, a formula was generated to calculate futile score and evaluate the risk of futile LT (*Fig. 3a*). The futile score had a good predictive capability with an AUC of 0.921 in the derivative cohort and 0.901 in the validation cohort (*Figs 3b* and *4a*). Comparative ROC analysis showed that the futile score performed better than the MELD and BAR scores. The calibration plot showed good calibration (*Figs 3c* and *4b*), and DCA revealed that the futile score had a higher net benefit (*Figs 3d and 4c*) in the derivation and validation cohorts.

**Table 2:** Univariable and multivariable logistic regression analysis of futile risk factors in futile group

	Futile (*n* = 69)	Not futile (*n* = 873)	Univariable analysis		Multivariable analysis	
			Odds ratio*	*P*	Odds ratio*	*P*
Donor age (years), mean(s.d.)	50.7(12.62)	46.33(13.04)	1.03 (1.01, 1.05)	0.008	1.05 (1.02, 1.08)	0.001
Donor BMI (kg/m^2^), mean(s.d.)	23.51(3.62)	23.46(2.80)	1.01 (0.92, 1.10)	0.886		
**Donor sex**				0.194		
Male	60 (87.0%)	703 (80.5%)	1.61 (0.78, 3.31)			
Female	9 (13.0%)	170 (19.5%)	1.00 (reference)			
Recipient age (years), mean(s.d.)	55.0(7.81)	49.23(10.73)	1.06 (1.03, 1.09)	< 0.001	1.116 (1.07, 1.17)	< 0.001
Recipient BMI (kg/m^2^), mean(s.d.)	22.66(2.95)	23.32(3.29)	0.94 (0.87, 1.01)	0.105		
**Recipient sex**						
Male	58 (84.1%)	713 (81.7%)	1.18 (0.61, 2.31)	0.621		
Female						
**Reason for transplantation**				0.349		
HBV/HCV	35 (50.8%)	480 (55.0%)	1.00 (reference)			
Alcoholic	25 (36.2%)	241 (27.6%)	1.42 (0.83, 2.43)	0.197		
Autoimmune	4 (5.8%)	44 (5.0%)	1.25 (0.42, 3.67)	0.689		
Other diseases	5 (7.2%)	108 (12.4%)	0.64 (0.24, 1.66)	0.354		
Malignancy	36 (52.2%)	350 (40.1%)	1.63 (0.10, 2.67)	0.051		
Hypertension	8 (11.6%)	96 (11.0%)	1.06 (0.49, 2.29)	0.879		
Diabetes mellitus	9 (13.0%)	90 (10.3%)	1.31 (0.63, 2.72)	0.477		
Portal vein thrombus	4 (5.8%)	31 (3.6%)	1.67 (0.57, 4.88)	0.347		
Previous TIPS	11 (15.9%)	77 (8.8%)	1.96 (0.99, 3.89)	0.054		
Previous abdominal surgery	9 (13.0%)	97 (11.1%)	1.20 (0.58, 2.49)	0.625		
Hepatic encephalopathy	8 (11.6%)	33 (3.8%)	3.34 (1.48, 7.54)	0.004	0.87 (0.27, 2.77)	0.811
Hepatorenal syndrome	24 (34.8%)	44 (5.0%)	10.05 (5.62, 17.96)	< 0.001	9.03 (4.11, 19.83)	< 0.001
ICU stay	32 (46.4%)	116 (13.3%)	5.64 (3.38, 9.42)	< 0.001	3.75 (1.51, 9.30)	0.004
Mechanical ventilator	14 (20.3%)	16 (1.8%)	13.63 (6.33-29.37)	< 0.001	6.95 (2.20, 22.00)	0.001
Artificial liver support	19 (27.5%)	75 (8.6%)	4.04 (2.27, 7.21)	< 0.001	2.55 (0.96, 6.72)	0.059
ABO incompatibility	32 (46.4%)	170 (19.5%)	3.58 (2.17, 5.91)	< 0.001	3.17 (1.60, 6.31)	0.001
DBD	51 (73.9%)	631 (72.3%)	0.92 (0.53, 1.61)	0.770		
CIT (h), mean(s.d.)	7.07(1.78)	5.95(1.97)	1.29 (1.15, 1.44)	< 0.001	1.48 (1.26, 1.73)	< 0.001
MELD score, mean(s.d.)	28.81(9.27)	21.98(10.91)	1.06 (1.04, 1.09)	< 0.001	1.11 (1.00, 1.24)	0.060
BAR score, mean(s.d.)	13.29(4.7)	8.46(5.53)	1.18 (1.12, 1.24)	< 0.001	0.84 (0.68, 1.05)	0.119
Pretransplant laboratory data						
WBC (× 10^9^/l), mean(s.d.)	8.06(4.95)	5.38(3.50)	1.52 (1.09, 1.21)	< 0.001	1.06 (0.99, 1.15)	0.115
Platelets (× 10^9^/l), mean(s.d.)	101.58(138.81)	88.54(68.45)	1.00 (0.99, 1.00)	0.176		
ALT (units/l), mean(s.d.)	187.25(232.46)	74.75(115.18)	1.00 (1.00, 1.00)	< 0.001	1.00 (1.00, 1.00)	0.032
AST (units/l), mean(s.d.)	189.28(203.92)	99.2(132.36)	1.00 (1.00, 1.00)	< 0.001	1.00 (0.99, 1.00)	0.883
Albumin (g/l), mean(s.d.)	32.15(5.85)	35.92(7.12)	0.92 (0.89, 0.96)	< 0.001	0.93 (0.88, 0.98)	0.006
Total bilirubin (mg/dl), mean(s.d.)	13.83(11.66)	6.9(7.89)	1.08 (1.05, 1.11)	< 0.001	1.04 (1.00, 1.08)	0.085
Plasma sodium (mmol/l), mean(s.d.)	137.74(7.79)	138.27(5.22)	0.98 (0.94, 1.03)	0.435		
Prothrombin time INR (s), mean(s.d.)	2.47(0.81)	2.11(0.67)	1.91 (1.41, 2.60)	< 0.001	0.61 (0.35, 1.09)	0.093

Values are *n* (%) unless otherwise stated: *values in parentheses are 95% confidence intervals. s.d., Standard deviation; BMI, body mass index; HBV, hepatitis B virus; HCV, hepatitis C virus; TIPS, transjugular intrahepatic portosystemic shunt; ICU, intensive care unit; DBD, donation after brain death; MELD, Model for End-stage Liver Disease; BAR, balance of risk; WBC, white blood cell; ALT, alanine aminotransferase; AST, aspartate aminotransferase; INR, international normalized ratio.

**Fig. 5:**
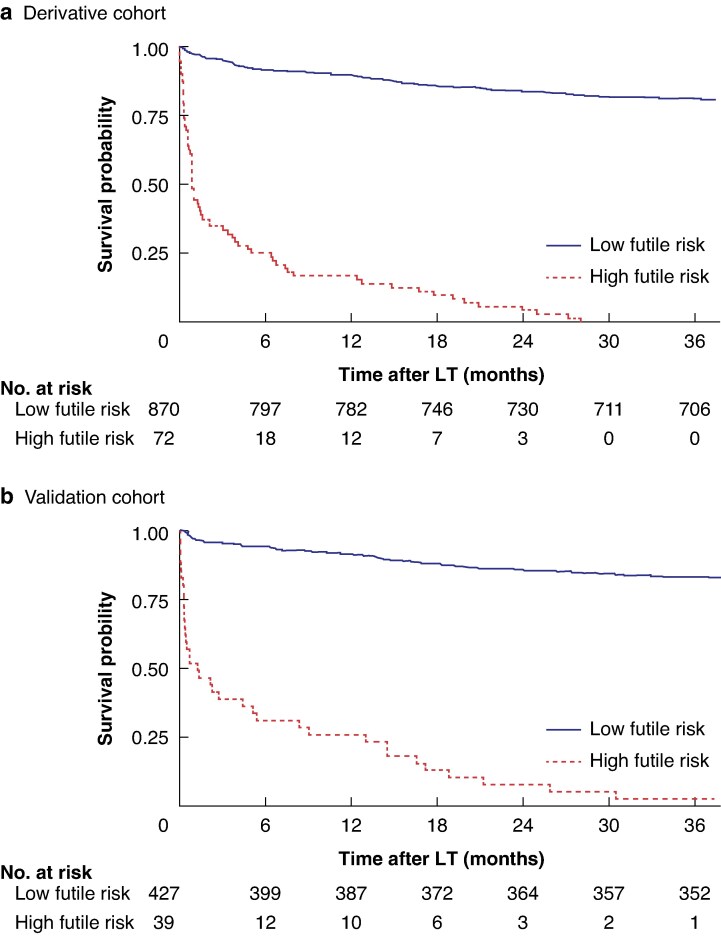
Survival analysis in relation to futile score Overall survival of groups with high (futile score ≥ 10.90) and low (< 10.90) futile risk in **a** derivative cohort and **b** validation cohort. **a**,**b**  *P* < 0.001 (log rank test).

Patients in the derivation and external cohorts were divided into groups with high (≥ 10.90) and low ( <10.90) futile risk based on the futile score according to the best cut-off, and the corresponding Kaplan–Meier survival curves were plotted (*Fig. 5*). In both cohorts, the group with high futile risk had significantly lower survival benefits (all *P* < 0.050), further confirming the value of clinical application of the futile scoring system.

## Discussion

In this study, multistage survival models were developed to track high-risk populations. Through survival analysis, it was found that only patients who were consistently in a high-risk state after LT could be considered truly futile for LT, whereas others should not be considered as such. Therefore, futile LT was redefined by multiple stages rather than a single pattern. Nine independent factors related to futile risk were delineated (older donor age, older recipient age, HRS, ICU stay, need for mechanical ventilator, ABO blood type incompatibility, prolonged CIT, increased ALT level, and decreased albumin level), and a formula was generated to calculate the futility score for evaluation of the risk of futile LT. The novel futile scoring system combining recipient and donor factors successfully outperformed other scoring systems, and effectively stratified the overall survival of the recipients according to the futile risk. This futile scoring system can be used to identify patients with a high futile risk before transplantation and provide appropriate treatment to improve their outcomes.

Despite some commonalities, the main risk factors for death at different stages after LT continued to show some heterogeneity. The present investigation revealed a significant correlation between patients’ individual conditions and surgical strategies with short- and mid-term survival outcomes after LT^17^. Extending to the long-term survival stage, apart from intrinsic patient factors, perioperative factors, graft quality, immunosuppressive regimen administration, and postoperative complications emerged as important risk factors, consistent with previous studies^18,19^. In light of the intricate and evolving nature of risk factors encountered after LT, the present authors devised survival models tailored to different stages, achieving commendable outcomes, which align with previous research^20,21^. These models, grounded in rigorous big-data analysis, incorporate patient demographics, clinical data, laboratory test results, and imaging parameters, to enhance the precision of risk prediction and evaluation^22^. The construction of survival models and stratification of risks by stage yielded an intriguing result: mortality risk is not static but rather dynamic. In this study, patients who faced a high mortality risk in the short term after LT could still attain long-term survival, if they successfully navigated the critical 3-month period with the aid of specific interventions. This result challenges the conventional single-stage approach to risk assessment, emphasizing the dynamic and changing nature of risk profiles^23^.

Ideally, accurately identifying futility would not only optimize the allocation of medical resources but also improve critical patient survival expectations^5,24^. However, the definition of futility is currently based on a single-stage assessment. In fact, for some critically ill patients, LT could offer long-term survival after specific treatment and care measures have been taken to overcome the early high-risk phase^25^. In this situation, it seems unfair to deny patients the opportunity to receive a liver transplant because of their high risk at a single stage being defined as futility, as this could deprive them of potential survival benefits. In the present study, rigorous survival analysis showed that, regardless of how risk groups were stratified, patients who remained at high risk across all three stages had the lowest survival benefit. Therefore, it is suggested that only patients who remain at high risk after LT should be considered in the truly futile group. In patients with poor outcomes, early identification and active treatment of risk factors associated with futility could prevent them from developing into futile transplantation and improve patients’ prognosis. This is also the basis for the authors’ study on whether or not to remove recipients from transplantation. The findings underscore the need for a more granular and individualized approach to risk management in the context of LT, driving both research efforts and clinical practice in the field of LT toward advances that prioritize personalized care and optimize patient outcomes^26^.

Notably, the present definition of futility was primarily grounded in the context of benign pathogeny, whereas the situation seemed to be different for patients with malignancy. After examination of the temporal evolution of pivotal mortality risk factors across various survival stages among the patients overall, a progressively intensifying role of malignancy was revealed over time. Regardless of the transplantation criteria, the risk of early postoperative death was not high for patients with malignancy, and most patients died from later tumour recurrence or metastasis^27^. Therefore, in line with previous research, the authors proposed that, for patients with an advanced cancer stage with extensive tumour load and/or macroscopic vascular invasion, long-term benefits from transplantation are difficult to achieve owing to poor prognosis^28^. However, some researchers disagreed, believing that, following successful downstaging treatment, LT conferred more survival advantages than the available alternative^29^. Historically, these patients have been denied transplantation because of organ shortages, among other reasons, but this seems to be changing in the era of living liver transplantation^30^.

The present investigation further elucidated futile LT, identifying several critical independent risk factors through multivariate analysis, such as older donor and recipient age, unstable recipient vital signs, ABO incompatibility, poorer recipient liver function, and prolonged CIT, which were appropriately reflected in the scoring system. The present study identified that having an older donor is a risk factor for graft failure, and older recipients have a higher risk of post-transplant mortality than younger patients^31,32^. Another study^33^ also found that unstable recipient vital signs and increasing recipient age were significant factors for poor outcomes. Furthermore, both ABO incompatibility and prolonged CIT can cause varying degrees of damage, leading to graft failure^34,35^. However, owing to the retrospective nature of the study, the risk factors and scoring system identified cannot be used as absolute criteria for all patients. Nevertheless, the futile risks calculated in this study should be considered, as well as more detailed indicators and trends of life-threatening organ failure, especially in terms of organ allocation and recipient selection.

A strength of this study, which contributed to refining the statistical rigour and improving the precision of the results, was that it excluded patients who died at a previous stage. Besides, it was found that patients with a high short-term mortality risk after LT did not necessarily have a high long-term risk because of the dynamic changes in mortality risk. The present study is unique in confirming that defining futility based on a single stage might reduce the survival expectations for certain patients.

Undoubtedly, this study has several limitations. First, this was a retrospective study, and there may be selection bias because all the patients enrolled underwent LT. Second, differences in the data collection protocols adopted by the different transplant centres may have influenced the predictive accuracy of the futile scoring system in different application contexts. A more comprehensive assessment of the generalizability of the models is therefore warranted. Finally, although the sample size was sufficient to support the findings of this study, there were limitations in translation to clinical practice owing to data availability and time constraints. To mitigate these concerns and further substantiate the present findings, larger-scale, prospective investigations are needed in the future.

In conclusion, the authors have redefined futile LT by building survival models to track high-risk populations by stage. The multiterm survival models constructed to predict the mortality risk of patients not only identified the main mortality risk factors in LT recipients, but also underscored notable temporal variations in these risk factors. Moreover, the findings indicate that exhibiting a high mortality risk in the short term after LT did not invariably portend a long-term high-risk profile, thereby necessitating a multistage, dynamic monitoring and assessment paradigm. This observation challenges the previous monolithic definition of futile LT, and reinforces the notion that only persistent high-risk status should be considered as truly futility. Based on the redefined futile LT, a novel visual futile scoring system was developed, which combined recipient and donor factors; this system successfully outperformed other scoring systems and effectively stratified overall survival of LT recipients according to futile risk. This approach could be used to identify the truly futile outcomes before LT, while optimizing the allocation of medical resources, especially in terms of organ allocation and recipient selection, providing a scientific rationale for developing personalized postoperative management strategies.

## Supplementary Material

zraf108_Supplementary_Data

## Data Availability

Data are available from the corresponding author upon reasonable request. The data are not publicly available as this could compromise the privacy of research participants.
