# Supplementary material for: Novel scoring system to predict futile liver transplantation by multiterm outcomes to optimize recipient selection: retrospective cohort study
Source: BJS Open. 2025 Oct 15;9(5):zraf108. doi: 10.1093/bjsopen/zraf108 (PMC12527355; doi:10.1093/bjsopen/zraf108)
Supplement: zraf108_Supplementary_Data [file zraf108_supplementary_data.docx]

**A novel scoring system to predict futile liver transplantation by multi-term outcomes to optimize recipient selection: retrospective cohort study**

Xi Wang ^1, a^ , Xiubi Yin ^1, a^, Shaohua Song ^2, a^, Di Jiang ^1^, Yuancheng Li ^1^, Zeliang Xu ^1^, Xingchao Liu ^3^, Zhu Li ^4^, Xiaofang Zhang ^1, *^, Chengcheng Zhang ^1, *^

^1^ Department of Hepatobiliary Surgery, Southwest Hospital, Third Military Medical University (Army Medical University), Shapingba District, Chongqing City, China

^2^ Department of General Surgery, Ruijin Hospital, Shanghai Jiao Tong University, School of Medicine, 197 Ruijin Second Road, Huangpu District, Shanghai, China 200025, China

^3^ Sichuan Academy of Medical Sciences & Sichuan Provincial People’s Hospital, Qingyang District, Sichuan Province, China

^4^ Department of Organ Transplantation Surgery, Liaocheng People's Hospital/Affiliated Liaocheng Hospital, Shandong First Medical University, 67 Dongchang West Road, Liaocheng, Shandong 252000, P.R. China

a These authors contributed equally to this article as co-first authors.

***Correspondence**

Xiaofang Zhang, Department of Hepatobiliary Surgery, Southwest Hospital, Third Military Medical University (Army Medical University), Shapingba District, Chongqing City, China.

E-mail: 572235882@qq.com;

Chengcheng Zhang, Department of Hepatobiliary Surgery, Southwest Hospital, Third Military Medical University (Army Medical University), Shapingba District, Chongqing City, China.

E-mail: [zhangcc@tmmu.edu.cn](mailto:zhangcc@tmmu.edu.cn).

**Supplementary Materials – Index**

**Supplementary Figures and Tables**

Fig. S1 Development and validation of survival models by stage in the derivative cohort. pag. 3

Fig. S2 Development and validation of survival models by stage in the validation cohort pag. 4

Fig. S3 Cause of futility in the futile group. pag. 5

Table S1 Univariate and multivariate logistic regression analysis of risk factors in the short-term survival group pag. 6-7

Table S2 Univariate and multivariate logistic regression analysis of risk factors in the mid-term survival group pag. 8-9

Table S3 Univariate and multivariate logistic regression analysis of risk factors in the long-term survival group pag.10-11


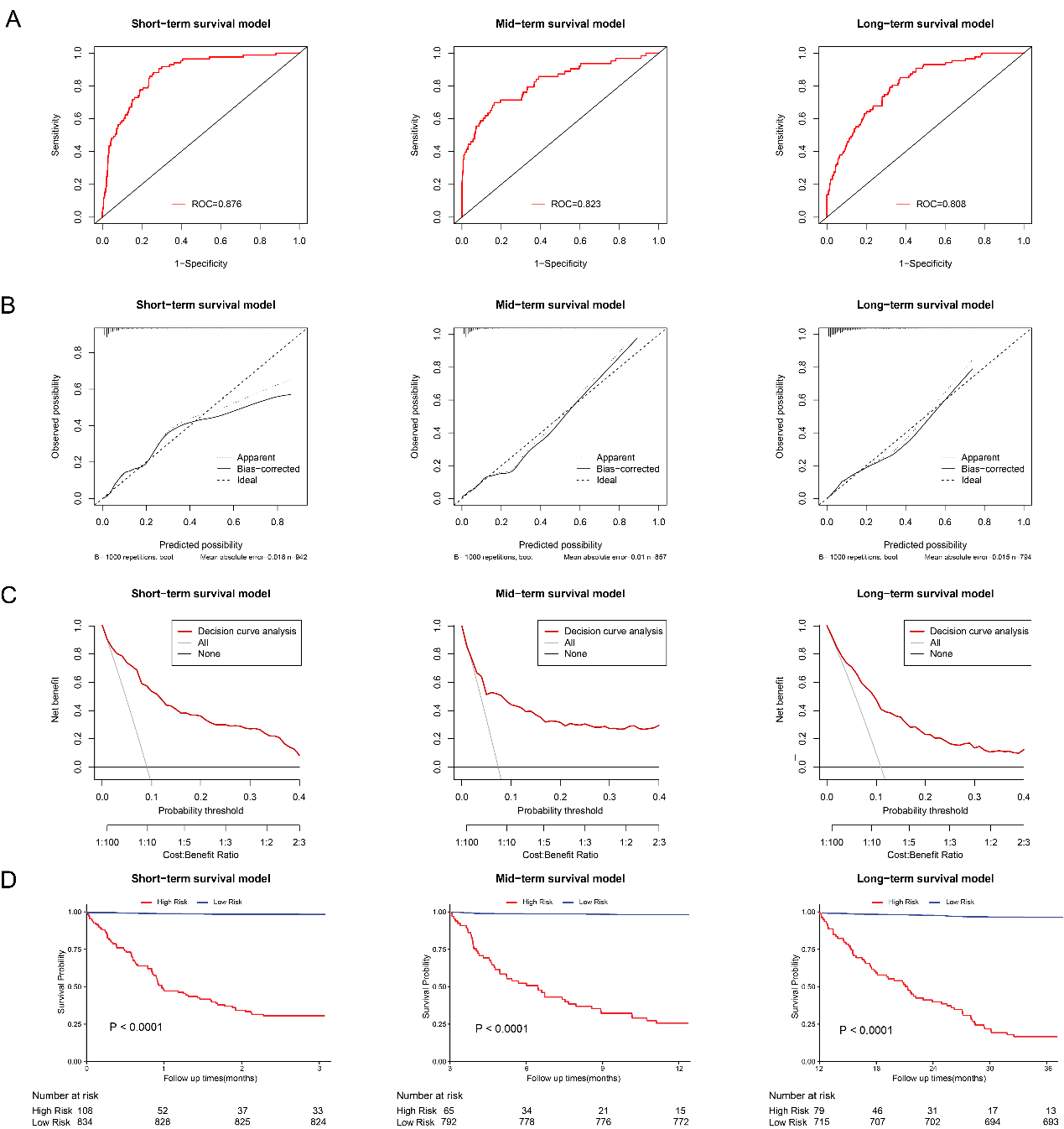


Fig. S1 Development and validation of survival models by stage in the derivative cohort. A ROC curves of short-term, mid-term and long-term survival models in the derivative cohort. B The corresponding calibration curve in the derivative cohort. C The DCA in the derivative cohort. D Kaplan–Meier curves for overall survival according to corresponding survival groups in the derivative cohort.


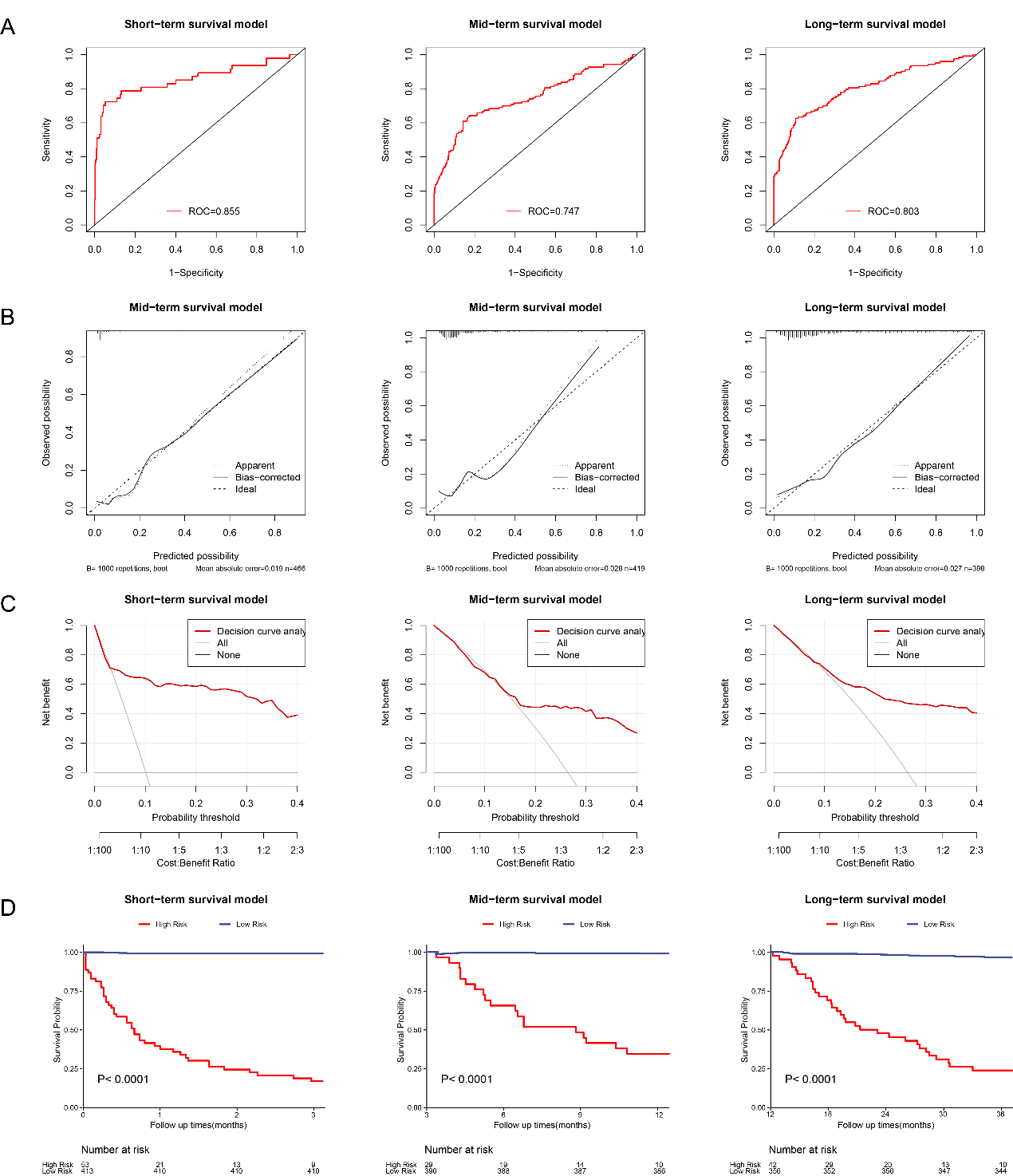


Fig. S2 Development and validation of survival models by stage in the validation cohort. A ROC curves of short-term, mid-term and long-term survival models in the validation cohort. B The corresponding calibration curve in the validation cohort. C The DCA in the validation cohort. D Kaplan–Meier curves for overall survival according to corresponding survival groups in the validation cohort.


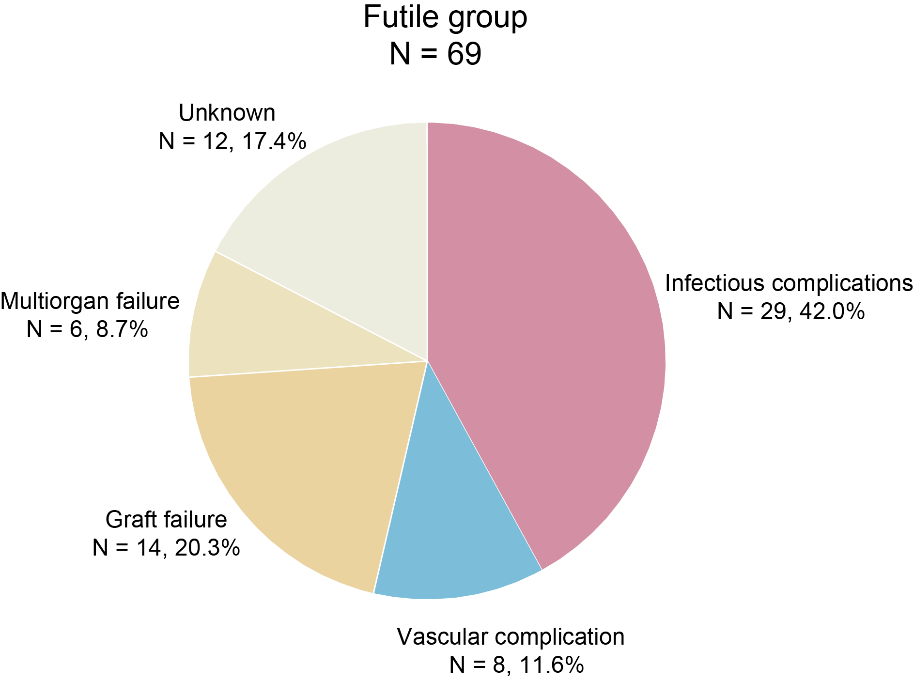


Fig. S3 Cause of futility in the futile group.

Table S1 Univariate and multivariate logistic regression analysis of risk factors in the short-term survival group

|  | Dead ≤ 3m  n = 85 | Alive > 3m  n= 857 | Univariate analysis | | Multivariate analysis | |
| --- | --- | --- | --- | --- | --- | --- |
|  |  |  | OR (95% CI) | P value | OR (95% CI) | P value |
| Donor Age, year | 44.13±13.16 | 46.9±13.02 | 0.984 (0.968-1.001) | 0.063 |  |  |
| Donor BMI, kg/ m^2^ | 23.37±3.43 | 23.48±2.81 | 0.987 (0.912-1.067) | 0.736 |  |  |
| Donor Sex, male | 69 (81.2) | 694 (81.0) | 1.013 (0.573-1.791) | 0.965 |  |  |
| Recipient Age, year | 52.34±8.66 | 49.39±10.79 | 1.028 (1.005-1.050) | 0.015 | 1.064 (1.031-1.099) | <0.001 |
| Recipient BMI, kg/ m^2^ | 23.14±3.65 | 23.29±3.24 | 0.986 (0.921-1.056) | 0.690 |  |  |
| Recipient Sex, male | 70 (82.4) | 701 (81.8) | 1.039 (0.579-1.862) | 0.899 |  |  |
| Cause of transplantation |  |  |  | 0.946 |  |  |
| HBV/HCV | 47 (55.3) | 468 (54.6) | Ref. |  |  |  |
| Alcoholic | 22 (25.9) | 244 (28.5) | 0.898 (0.529-1.524) |  |  |  |
| Autoimmune | 5 (5.9) | 43 (5.0) | 1.158 (0.437-3.065) |  |  |  |
| Other diseases | 11 (12.9) | 102 (11.9) | 1.074 (0.538 -2.142) |  |  |  |
| Malignancy | 29 (34.1) | 357 (41.7) | 0.725 (0.454-1.159) | 0.179 |  |  |
| HTN | 17 (20.0) | 87 (10.2) | 2.213 (1.244-3.936) | 0.007 | 1.432 (0.695-2.951) | 0.331 |
| DM | 11 (12.9) | 88 (10.3) | 1.299 (0.664-2.540) | 0.445 |  |  |
| PVT | 6 (7.1) | 29 (3.4) | 2.168 (0.874-5.381) | 0.095 |  |  |
| Previous TIPS | 14 (16.5) | 74 (8.6) | 2.086 (1.122-3.881) | 0.020 | 2.351 (1.020-5.418) | 0.045 |
| Previous abdominal surgery | 13 (15.3) | 93 (10.9) | 1.483 (0.791-2.781) | 0.219 |  |  |
| Hepatic encephalopathy | 8 (9.4) | 33 (3.9) | 2.594 (1.158-5.814) | 0.021 | 0.895 (0.315-2.540) | 0.834 |
| HRS | 22 (25.9) | 46 (5.4) | 6.157 (3.485-10.876) | <0.001 | 4.960 (2.476-9.940) | <0.001 |
| ICU stay | 39 (45.9) | 109 (12.7) | 5.818 (3.631-9.324) | <0.001 | 4.082 (1.869-8.915) | <0.001 |
| Mechanical ventilator | 16 (18.8) | 14 (1.6) | 13.963 (6.542-29.799) | <0.001 | 4.832 (1.756-13.296) | 0.002 |
| Artificial liver support | 27 (31.8) | 67 (7.8) | 5.489 (3.262-9.235) | <0.001 | 4.005 (1.804-8.891) | 0.001 |
| ABO incompatibility | 35 (41.2) | 167 (19.5) | 2.892 (1.819-4.599) | <0.001 | 1.973 (1.123-3.465) | 0.018 |
| DBD | 26 (30.6) | 234 (27.3) | 1.173 (0.722-1.906) | 0.519 |  |  |
| CIT, h | 6.89±1.85 | 5.95±1.97 | 1.244 (1.120-1.381) | <0.001 | 1.395 (1.222-1.592) | <0.001 |
| MELD score | 28.81±10.08 | 21.86±10.83 | 1.062 (1.039-1.085) | <0.001 | 1.195(1.092-1.309) | <0.001 |
| BAR score | 12.67±5.29 | 8.43±5.5 | 1.151 (1.102-1.203) | <0.001 | 0.739 (0.619-0.882) | 0.001 |
| Pre-transplant Laboratory |  |  |  |  |  |  |
| WBC, × 10^9^/L | 7.40±5.11 | 5.40±3.47 | 1.119 (1.066-1.175) | <0.001 | 1.031 (0.996-1.100) | 0.361 |
| PLT, × 10^9^/L | 94.75±127.42 | 88.97±68.70 | 1.001 (0.998-1.004) | 0.503 |  |  |
| ALT, IU/L | 104.00±156.52 | 80.91±127.67 | 1.001 (1.000-1.003) | 0.120 |  |  |
| AST, IU/L | 122.41±139.47 | 104.16±140.78 | 1.001 (0.999-1.002) | 0.256 |  |  |
| ALB, g/L | 33.53±6.74 | 35.85±7.10 | 0.952 (0.921-0.985) | 0.004 | 0.984 (0.943-1.026) | 0.436 |
| TBIL, mg/dL | 12.83±11.35 | 6.87±7.87 | 1.069 (1.046-1.094) | <0.001 | 1.023 (0.986-1.061) | 0.221 |
| Na, mmol/L | 136.66±6.82 | 138.38±5.27 | 0.945 (0.908-0.983) | 0.005 | 0.965 (0.924-1.007) | 0.100 |
| PT-INR, s | 2.48±0.87 | 2.11±0.66 | 1.961 (1.479-2.599) | <0.001 | 0.807 (0.517-1.258) | 0.344 |

*BMI* body mass index; *HBV* hepatitis B virus; *HCV* hepatitis C virus; *HTN* hypertension; *DM* diabetes mellitus; *PVT* portal vein thrombus; *TIPS* previous transjugular intrahepatic portosystemic shunt; *HRS* hepatorenal syndrome; *ICU* intensive care unit; *DBD* donation after brain death; *CIT* cold ischemia time; *MELD* the model for end-stage liver disease; *BAR* balance of risk; *WBC* white blood cell; *PLT* platelet; *ALT* alanine aminotransferase; *AST* aspartate aminotransferase; *ALB* albumin; *TBIL* total bilirubin; *Na* plasma sodium; *PT-INR* prothrombin time international normalized ratio

Table S2 Univariate and multivariate logistic regression analysis of risk factors in the mid-term survival group

|  | 3m< Dead ≤ 1y  n =63 | Alive > 1y  n= 794 | Univariate analysis | | Multivariate analysis | |
| --- | --- | --- | --- | --- | --- | --- |
|  |  |  | OR (95% CI) | P value | OR (95% CI) | P value |
| Donor Age, year | 46.49±13.57 | 46.93±12.99 | 0.997 (0.978-1.017) | 0.799 |  |  |
| Donor BMI, kg/ m^2^ | 23.49±2.55 | 23.47±2.83 | 1.003 (0.915-1.099) | 0.955 |  |  |
| Donor Sex, male | 51 (81.0) | 643 (81.0) | 0.998 (0.519-1.918) | 0.995 |  |  |
| Recipient Age, year | 50.25±9.82 | 49.32±10.87 | 1.008 (0.984-1.033) | 0.507 |  |  |
| Recipient BMI, kg/ m^2^ | 23.95±3.42 | 23.23±3.22 | 1.069 (0.989-1.154) | 0.092 |  |  |
| Recipient Sex, male | 50 (79.4) | 651 (81.0) | 0.845 (0.447-1.597) | 0.604 |  |  |
| Cause of transplantation |  |  |  | 0.022 |  | 0.080 |
| HBV/HCV | 28 (44.4) | 440 (55.4) | Ref. |  | Ref. |  |
| Alcoholic | 27 (42.9) | 217 (27.3) | 1.955 (1.125-3.399) | 0.017 | 1.763(0.884-3.518) | 0.108 |
| Autoimmune | 5 (7.9) | 38 (4.8) | 2.068 (0.755-5.664) | 0.158 | 3.481(1.079-11.232) | 0.037 |
| Other diseases | 3 (4.8) | 99 (12.5) | 0.476 (0.142-1.598) | 0.230 | 0.843(0.206-3.448) | 0.812 |
| Malignancy | 36 (57.1) | 321 (40.4) | 1.965 (1.170-3.300) | 0.011 | 2.371(1.139-4.937) | 0.021 |
| HTN | 10 (15.9) | 77 (9.7) | 1.757 (0.859-3.593) | 0.123 |  |  |
| DM | 6 (9.5) | 82 (10.3) | 0.914 (0.382-2.185) | 0.840 |  |  |
| PVT | 1 (1.6) | 28 (3.5) | 0.441 (0.059-3.298) | 0.425 |  |  |
| Previous TIPS | 4 (6.3) | 70 (8.8) | 0.701 (0.247-1.988) | 0.504 |  |  |
| Previous abdominal surgery | 6 (9.5) | 87 (11.0) | 0.855 (0.358-2.042) | 0.725 |  |  |
| Hepatic encephalopathy | 7 (11.1) | 26 (3.3) | 3.692 (1.535-8.880) | 0.004 | 5.602(1.682-18.657) | 0.005 |
| HRS | 8 (12.7) | 38 (4.8) | 2.894 (1.287-6.505) | 0.010 | 1.191(0.396-3.584) | 0.756 |
| ICU stay | 11 (17.5) | 98 (12.3) | 1.502 (0.758-2.977) | 0.243 |  |  |
| Mechanical ventilator | 3 (4.8) | 11 (1.4) | 3.559 (0.967-13.013) | 0.056 |  |  |
| Artificial liver support | 7 (11.1) | 60 (7.6) | 1.529 (0.668-3.502) | 0.315 |  |  |
| ABO incompatibility | 13 (20.6) | 154 (19.4) | 1.081 (0.573-2.039) | 0.811 |  |  |
| DBD | 13 (20.6) | 221 (27.8) | 0.674 (0.359-1.265) | 0.220 |  |  |
| CIT, h | 6.54±1.96 | 5.9±1.96 | 1.162 (1.031-1.310) | 0.014 | 1.21(1.041-1.407) | 0.013 |
| MELD score | 24.33±11.73 | 21.66±10.74 | 1.023 (0.999-1.047) | 0.061 |  |  |
| BAR score | 9.95±5.65 | 8.31±5.47 | 1.055 (1.007-1.105) | 0.024 | 0.996(0.933-1.063) | 0.902 |
| Pre-transplant Laboratory |  |  |  |  |  |  |
| WBC, × 10^9^/L | 6.78±4.8 | 5.29±3.32 | 1.104 (1.039-1.172) | 0.001 | 0.984(0.902-1.073) | 0.714 |
| PLT, × 10^9^/L | 104.37±91.2 | 87.75±66.51 | 1.003 (1.000-1.006) | 0.067 |  |  |
| ALT, IU/L | 273.26±272.64 | 65.65±92.79 | 1.006 (1.005-1.008) | <0.001 | 1.006(1.003-1.009) | <0.001 |
| AST, IU/L | 285.15±239.85 | 89.79±118.68 | 1.005 (1.005-1.007) | <0.001 | 1.002(0.999-1.005) | 0.140 |
| ALB, g/L | 33.66±5.65 | 36.02±7.18 | 0.952 (0.917-0.989) | 0.011 | 0.922(0.878-0.969) | 0.001 |
| TBIL, mg/dL | 9.72±10.15 | 6.65±7.62 | 1.043 (1.014-1.072) | 0.003 | 1.055(1.01-1.102) | 0.016 |
| Na, mmol/L | 138.66±4.96 | 138.36±5.29 | 1.011 (0.963-1.061) | 0.671 |  |  |
| PT-INR, s | 2.3±0.82 | 2.09±0.64 | 1.544 (1.092-2.184) | 0.014 | 0.409(0.222-0.754) | 0.004 |

*BMI* body mass index; *HBV* hepatitis B virus; *HCV* hepatitis C virus; *HTN* hypertension; *DM* diabetes mellitus; *PVT* portal vein thrombus; *TIPS* previous transjugular intrahepatic portosystemic shunt; *HRS* hepatorenal syndrome; *ICU* intensive care unit; *DBD* donation after brain death; *CIT* cold ischemia time; *MELD* the model for end-stage liver disease; *BAR* balance of risk; *WBC* white blood cell; *PLT* platelet; *ALT* alanine aminotransferase; *AST* aspartate aminotransferase; *ALB* albumin; *TBIL* total bilirubin; *Na* plasma sodium; *PT-INR* prothrombin time international normalized ratio

Table S3 Univariate and multivariate logistic regression analysis of risk factors in the long-term survival group

|  | 1y< Dead ≤ 3y  n = 87 | Alive > 3y  n= 707 | Univariate analysis | | Multivariate analysis | |
| --- | --- | --- | --- | --- | --- | --- |
|  |  |  | OR (95% CI) | P value | OR (95% CI) | P value |
| Donor Age, year | 50±14.43 | 46.55±12.76 | 1.022 (1.003-1.040) | 0.020 | 1.026(1.005-1.046) | 0.012 |
| Donor BMI, kg/ m^2^ | 23.43±2.49 | 23.48±2.87 | 0.994 (0.918-1.075) | 0.875 |  |  |
| Donor Sex, male | 71 (81.6) | 572 (80.9) | 1.047 (0.590-1.859) | 0.875 |  |  |
| Recipient Age, year | 52.99±10.88 | 48.86±10.79 | 1.038 (1.015-1.061) | 0.001 | 1.057(1.031-1.084) | <0.001 |
| Recipient BMI, kg/ m^2^ | 21.84±3.46 | 23.4±3.15 | 0.848 (0.785-0.915) | <0.001 | 0.805(0.738-0.878) | <0.001 |
| Recipient Sex, male | 71 (81.6) | 580 (82.0) | 0.972 (0.547-1.727) | 0.922 |  |  |
| Cause of transplantation |  |  |  | 0.229 |  |  |
| HBV/HCV | 49 (56.3) | 391 (55.3) | Ref. |  |  |  |
| Alcoholic | 28 (32.2) | 189 (26.7) | 1.182 (0.720-1.941) | 0.508 |  |  |
| Autoimmune | 5 (5.7) | 33 (4.7) | 1.209 (0.451-3.242) | 0.706 |  |  |
| Other diseases | 5 (5.7) | 94 (13.3) | 0.424 (0.165-1.095) | 0.076 |  |  |
| Malignancy | 54 (62.1) | 267 (37.8) | 2.697 (1.704-4.267) | <0.001 | 3.627(2.108-6.242) | <0.001 |
| HTN | 6 (6.9) | 71 (10.0) | 0.664 (0.279-1.576) | 0.353 |  |  |
| DM | 8 (9.2) | 74 (10.5) | 0.866 (0.403-1.863) | 0.713 |  |  |
| PVT | 1 (1.1) | 27 (3.8) | 0.293 (0.039-2.182) | 0.231 |  |  |
| Previous TIPS | 6 (6.9) | 64 (9.1) | 0.744 (0.312-1.773) | 0.505 |  |  |
| Previous abdominal surgery | 9 (10.3) | 78 (11.0) | 0.930 (0.449-1.929) | 0.846 |  |  |
| Hepatic encephalopathy | 3 (3.4) | 23 (3.3) | 1.062 (0.312-3.613) | 0.923 |  |  |
| HRS | 5 (5.7) | 33 (4.7) | 1.245 (0.473-3.279) | 0.657 |  |  |
| ICU stay | 13 (14.9) | 85 (12.0) | 1.286 (0.684-2.417) | 0.436 |  |  |
| Mechanical ventilator | 1 (1.1) | 10 (1.4) | 0.810 (0.102-6.409) | 0.842 |  |  |
| Artificial liver support | 10 (11.5) | 50 (7.1) | 1.706 (0.832-3.502) | 0.145 |  |  |
| ABO incompatibility | 29 (33.3) | 125 (17.7) | 2.328 (1.432-3.784) | 0.001 | 2.166(1.246-3.764) | 0.006 |
| DBD | 75 (86.2) | 498 (70.4) | 0.381 (0.203-0.716) | 0.003 | 0.390(0.196-0.776) | 0.007 |
| CIT, h | 6.49±2.3 | 5.83±1.91 | 1.170 (1.052-1.301) | 0.004 | 1.228(1.087-1.388) | 0.001 |
| MELD score | 22.7±10.66 | 21.53±10.75 | 1.010 (0.990-1.031) | 0.338 |  |  |
| BAR score | 9.32±5.93 | 8.19±5.41 | 1.038 (0.997-1.081) | 0.069 |  |  |
| Pre-transplant Laboratory |  |  |  |  |  |  |
| WBC, × 10^9^/L | 6.36±4.11 | 5.15±3.19 | 1.096 (1.035-1.161) | 0.002 | 1.104(1.028-1.187) | 0.007 |
| PLT, × 10^9^/L | 87.71±70.04 | 87.76±66.12 | 1.000 (0.997-1.003) | 0.995 |  |  |
| ALT, IU/L | 61.02±88.95 | 66.22±93.3 | 0.999 (0.997-1.002) | 0.623 |  |  |
| AST, IU/L | 81.08±92.32 | 90.86±121.54 | 0.999 (0.997-1.001) | 0.469 |  |  |
| ALB, g/L | 35.85±7.49 | 36.05±7.14 | 0.996 (0.966-1.028) | 0.812 |  |  |
| TBIL, mg/dL | 8.94±9.77 | 6.36±7.27 | 1.039 (1.013-1.066) | 0.003 | 1.050(1.017-1.084) | 0.003 |
| Na, mmol/L | 138.03±5.41 | 138.41±5.28 | 0.987 (0.946-1.029) | 0.527 |  |  |
| PT-INR, s | 2.08±0.62 | 2.09±0.64 | 0.972 (0.684-1.380) | 0.873 |  |  |

*BMI* body mass index; *HBV* hepatitis B virus; *HCV* hepatitis C virus; *HTN* hypertension; *DM* diabetes mellitus; *PVT* portal vein thrombus; *TIPS* previous transjugular intrahepatic portosystemic shunt; *HRS* hepatorenal syndrome; *ICU* intensive care unit; *DBD* donation after brain death; *CIT* cold ischemia time; *MELD* the model for end-stage liver disease; *BAR* balance of risk; *WBC* white blood cell; *PLT* platelet; *ALT* alanine aminotransferase; *AST* aspartate aminotransferase; *ALB* albumin; *TBIL* total bilirubin; *Na* plasma sodium; *PT-INR* prothrombin time international normalized ratio
